# Fatal 3-Nitropropionic Acid Poisoning after Consuming Coconut Water

**DOI:** 10.3201/eid2701.202222

**Published:** 2021-01

**Authors:** Thomas Birkelund, Rakel F. Johansen, Dorte G. Illum, Stig Eric Dyrskog, Jakob A. Østergaard, Travis M. Falconer, Chris Andersen, Helena Fridholm, Søren Overballe-Petersen, Jørgen S. Jensen

**Affiliations:** Prehospital Emergency Medical Services, Central Denmark Region, Denmark (T. Birkelund);; Aarhus University Hospital, Aarhus, Denmark (T. Birkelund, R.F. Johansen, D.G. Illum, S.E. Dyrskog, J.A. Østergaard);; US Food and Drug Administration, Cincinnati, Ohio, USA (T.M. Falconer); Aarhus University, Aarhus (C. Andersen);; Statens Serum Institut, Copenhagen, Denmark (H. Fridholm, S. Overballe-Petersen, J.S. Jensen)

**Keywords:** poisoning, 3-nitropropionic acid, coconut, fungi, Arthrinium saccharicola

## Abstract

We describe the fatal course of a patient with initial symptoms of vomiting and nausea who developed symptoms of dystonia, encephalopathy, and coma. The cause of death was poisoning with 3-nitropropionic acid from coconut water spoiled with the fungus *Arthrinium saccharicola*. We present the clinical findings and forensic analysis.

A 69-year old Caucasian man was admitted to Aarhus University Hospital, Aarhus, Denmark, in a state of reduced consciousness and Glasgow Coma Scale (GCS) score of 13–14. Approximately 4.5 hours before admission, the patient had consumed coconut water directly from a coconut using a straw. Because the water had a foul taste, he swallowed only a small amount. Afterward, he opened the nut and described to his wife that the interior was slimy and looked rotten.

The coconut was preshaved, with visible endosperm (coconut meat) at the top for easy access to the carpels (holes) and the coconut water. A straw was included and used for puncturing the coconut at the time of consumption (Figure). Recommended storage was at 4°C–5°C in the refrigerator, but the coconut had been kept on the kitchen table for 1 month after purchase.

**Figure:**
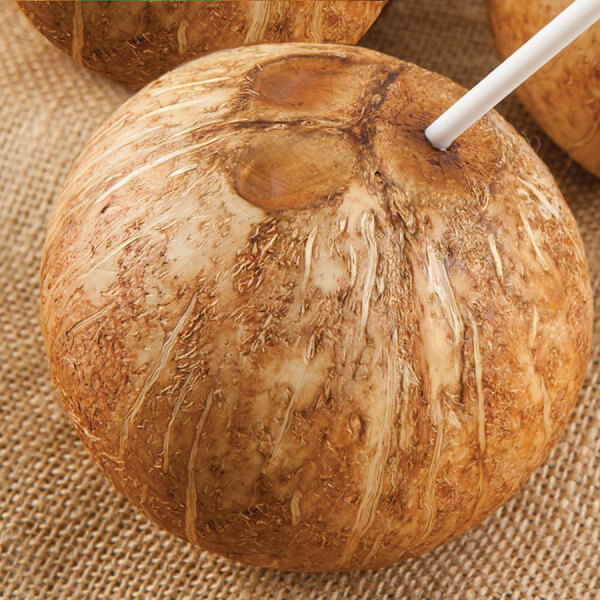
The type of ecologically grown coconut involved in the case of a 69-year old Caucasian man in Aarhus, Denmark, who died of poisoning with 3-nitropropionic acid from coconut water spoiled with the fungus *Arthrinium saccharicola*. The coconut was commercially prepared, including removal of the husk, and was sold as ready-to-drink, with an included punch and straw for easy access to the carpels (holes) and the coconut water.

Approximately 3 hours after drinking the coconut water, the patient developed sweating, nausea, and vomiting. Ambulance attendants found the patient in distress with pale and clammy skin, reduced mental state with confusion, dystonia, and poor balance but normal strength in the extremities. The patient was able to answer questions with delay and had no local neurologic deficits.

At the emergency department, the patient’s consciousness level descended to GCS 3. A computed tomography angiography of the cerebrum, thorax, abdomen, and pelvis was performed and showed no abnormalities. Blood pressure was 200/110 mm Hg; pupils were small and reactive to light. The physical exam, including the abdomen, was unremarkable. An electrocardiogram showed sinus tachycardia of 130 beats/min with new right bundle branch block, which had not been present on an electrocardiogram 7 months earlier. Body temperature was 37.5°C.

Arterial blood gas analysis revealed metabolic acidosis with a pH of 7.29, PaCO_2_ of 2.1 kPa (reference 9.6–13.7 kPa), base excess −18.3 mmol/L (reference −1.5 to –3.0 mmol/L), lactate level of 10.9 mmol/L (reference 0.5–2.5 mmol/L), and unremarkable strong ion difference. Elevated 3-hydroxybutyrate level of 2.2 mmol/L (reference <0.3 mmol/L) and slightly elevated blood glucose of 9.5 mmol/L (reference 4.2–7.8 mmol/L) were found. Other initial blood samples were unremarkable. Carbon monoxide was not detected.

The patient was brought to the intensive care unit 2 hours after his arrival at the hospital. At this point he had pronation and jerks of the forearms and calves and his body temperature had increased to 39.7°C. The patient was intubated and actively cooled to 37.5°C. Treatment for meningitis and encephalitis (penicillin, ceftriaxone, and acyclovir) was initiated and insulin was administered, keeping blood glucose at 5–10 mmol/L. Pinpoint pupils were noted; to rule out opioid intoxication, naloxone was administered, with no response. The patient had stable hemodynamics at the intensive care unit. High diuresis of <450 mL/hour was noticed. Interviews with the family provided no reason to suspect intake of acetaminophen, salicylic acid, methanol, glycols, or other acids or drugs or a suicide attempt. A urine drug screen was negative except for opioids, which had been administered to facilitate intubation.

Lumbar puncture was performed 11 and 22 hours after admission. No blood, leukocytes, or protein were detected. PCR examination for bacteria and herpes encephalitis, including cultures, was negative. EEG showed nonspecific abnormalities without seizure activity or reactivity on stimulation.

Fourteen hours after admission, magnetic resonance imaging of the cerebrum demonstrated global diffusion restrictions in the white matter. T2-weighted fluid-attenuated inversion recovery signal showed edema in the putamen, hippocampi, and cerebellum. Judging by the magnetic resonance imaging findings, severe toxic/metabolic encephalopathy was suspected. Sixteen hours after admission, new blood samples revealed levels of ammonia 100 μmol/L (reference <50 μmol/L), myoglobin 1,635 μg/L (reference <75 μg/L) and creatine kinase 905 U/L (reference 50–200 U/L). Approximately 24 hours after admission, a new computed tomography scan of the cerebrum showed severe edema, especially in the infratentorial region, with impending signs of brain herniation. The pupils were dilated, brainstem reflexes were absent, and the patient had no spontaneous respiration. The patient was not sedated at any point during treatment. Twenty-six hours after admission to the hospital, the clinical appearance and imaging indicated clinical brain death, and treatment was discontinued.

Medicolegal autopsy of the cerebrum showed sporadic microscopic bleedings in the basal ganglia. Analysis of tracheal aspirate revealed growth of the fungus *Rhizopus arrhizus*. Because the patient developed symptoms after intake of a coconut product, intoxication with bongkrekic acid was suspected. This mitochondrial toxin is produced by the bacterium *Burkholderia gladioli* pathovar cocovenenans (*B. cocovenenans*) and has been implicated in outbreaks of foodborne illness involving coconut products in Asia (*1*). We therefore analyzed a part of the coconut. Culture revealed growth of *Pseudomonas* species and *R. arrhizus.* Because some *Rhizopus* spp. carry *Burkholderia* spp. as symbionts (*2*), we subjected DNA from the fungal isolate to PCR with primers targeting universal bacterial 16S rRNA gene sequences, but no amplification of bacterial DNA was seen.

We subjected DNA extracted from the coconut to microbiota characterization with Illumina sequencing (https://www.illumina.com) of 16S and 18S rRNA gene products amplified with universal primers (*3*); among eukaryotic 18S sequences, most were identified as *Arthrinium* spp. and *R. arrhizus*, which were isolated from the coconut. Most bacterial 16S sequences were mapped to *Pseudomonas fragi*, a common spoilage bacterium. No sequences were mapped to *Burkholderia* spp.

Because of the strong suspicion of bongkrekic acid, we tested extracted DNA with 3 *Burkholderia*-specific PCR assays (developed for differentiation of *B. cocovenenans* from other species [*4*]); all results were negative. We sent pieces of the coconut endosperm for detection of bongkrekic acid to the US FDA Forensic Chemistry Center (Cincinnati, Ohio, USA), which has developed a method for the detection of bongkrekic acid (*5*,*6*). However, neither bongkrekic acid nor the isomer isobongkrekic acid could be detected at a level of >4 μg/g.

We instilled homogenized coconut via gastric tube in a set of 3 mice, but no toxicity was observed, even after prolonged observation. Metagenomic sequencing using MinION sequencing (Oxford Nanopore Technologies, https://nanoporetech.com) initially revealed only *Pseudomonas* species using Oxford Nanopore’s online tools. However, upon further analysis with the tool K-Mer Aligner (*7*), *Arthrinium saccharicola* was mapped at 145× coverage, *Arthrinium* mitochondria at 180×, and *Pseudomonas* sp*.* at 290×.

## Conclusions

Fungi of the *Arthrinium* genus produce the lipophilic and highly toxic 3-nitropropionic acid (3-NPA), which is involved in the etiology of moldy sugar cane poisoning (*8*) with severe encephalopathy (*9*). 3-NPA irreversibly binds to and inhibits succinate dehydrogenase in the mitochondria, thereby blocking the citric acid cycle and ATP generation in cells, which would explain the observed severe lactate acidosis. Other proposed mechanisms include increased generation of reactive oxygen species and release of apoptogenic factors in the cytosol of the basal ganglia, resembling the pathological findings and clinical symptoms related to Huntington’s disease (*10*,*11*).

We reexamined the coconut endosperm and a blood sample from the patient for 3-NPA, which was detected at level of ≈120 mcg/g in the coconut sample and 0.36 mcg/g in blood from the patient. The oral lethal dose for mice is 68 mcg/g (Sigma safety data sheet, https://pubchem.ncbi.nlm.nih.gov/compound/1678), but the toxic dose for humans is not known, and rodents are likely to be more resistant to toxicity, in agreement with the lack of symptoms in the mouse experiment. The symptoms of 3-NPA toxicity in humans are similar to those for bongkrekic acid, as described regarding sugar cane poisoning in humans in China and Africa, including initial gastrointestinal symptoms with vomiting and diarrhea and progressing encephalopathy leading to coma and death (*8*,*11*,*12*). These symptoms are similar to those of the patient. The collaboration between several national and international authorities contributed to resolve this challenging case, providing an understanding of the rapid disease progression and sudden death of the patient.
